# “I’ve Never Really Thought about This…” – The Complexity of Patient Involvement in Education from the Teachers’ Point of View

**DOI:** 10.5334/pme.1875

**Published:** 2026-03-05

**Authors:** Elias Schriwer, Agnes Elmberger, Anders Sondén, Terese Stenfors

**Affiliations:** 1 PhD student, Department of Learning, Informatics, Management and Education, Karolinska Institutet, Stockholm, Sweden; 2 Researcher, Department of Learning, Informatics, Management and Education, Karolinska Institutet, Stockholm, Sweden; 3 Associate Professor, Department of Clinical Science and Education, Södersjukhuset, Stockholm, Sweden; 4 Professor, Department of Learning, Informatics, Management and Education, Karolinska Institutet, Stockholm, Sweden

## Abstract

**Introduction:**

Patient involvement in health professions education (HPE) often takes the form of isolated, small-scale activities, where teachers’ initiatives largely determine whether and how patients are included. Understanding how teachers make sense of patient involvement is therefore crucial for strengthening and sustaining this practice in HPE. Using a phenomenographic approach, our aim was to explore how teachers understand patient involvement in HPE and visualize these interrelated understandings in an outcome space.

**Methods:**

We conducted semi-structured interviews with 20 teachers experienced in working with patient involvement across eight different HPE programs at one university. The interview guide was developed within the research group, in dialogue with a patient representative.

**Results:**

We identified four qualitatively different ways in which teachers understand patient involvement: as a way to 1) create variation in education, 2) improve education, 3) improve health care or 4) improve society. These four categories were comprised of seven themes of expanding awareness: the role of the patient, the role of the student, the role of the teacher, role of education, interaction between patient and student, view of workplace learning and teachers’ perceived agency.

**Discussion and conclusion:**

Our findings suggest that there is considerable complexity in how teachers understand the purpose and value of patient involvement in education. These differences will influence why, how, and to what extent patients are involved in HPE. Striving towards a more complex understanding may support more beneficial, sustainable and meaningful patient involvement. Further research should explore how different stakeholders’ understandings influence the practice of patient involvement in HPE.

## Introduction

The patient is the core of health care and patient-centered approaches are necessary for improving quality and outcomes [1,2]. To achieve patient-centered care, health professionals need to understand the patient’s perspective and there have been previous calls to increase the inclusion of patients, not only in healthcare, but also in education [3,4,5].

Patients have, in some sense, always been involved in health professions education (HPE) for example through patient demonstrations [6,7], but more recently, patients are being involved in an increasing number of ways, such as teachers, assessors and in administrative leadership roles [8,9,10,11,12]. Frameworks are now available to make structured implementation of patient involvement easier, as well as to create a sustainable environment for collaboration with patients [8,13,14,15,16]. Several benefits of patient involvement have been identified including increased learning and meaningfulness [11,13,17,18]. Concerns about patient involvement in HPE have also been explored such as representation and teacher prejudice [19,20].

Despite the identified benefits, patient involvement in HPE is still often an isolated, ad-hoc event and it is reasonable to assume that teachers play a crucial role in the implementation of patient involvement in HPE [9,14,15,21,22]. Rowland et al propose an exploration of the driving forces that motivate each stakeholder working with patient involvement [22] as understanding these is key to achieving structured and sustainable collaboration across all groups (teachers, patients and students). Recent literature has started exploring these ideas but currently, we lack research on what patient involvement means for teachers specifically and why it may be of importance to them [21,23]. If frameworks do not relate to a key stakeholder’s understanding and reasoning behind patient involvement in HPE, their use might be in vain. Understanding how teachers make sense of patient involvement is therefore critical, not only because teachers are key actors in implementation, but because their underlying reasoning shapes the logic through which patient involvement is prioritized, sustained, and legitimized within HPE.

To help explore teachers’ perceptions of patient involvement in HPE, we draw primarily on phenomenography as our methodological and epistemological approach [24]. The development of phenomenography has been shaped by several psychological traditions: Piagetian psychology, which highlighted qualitative differences in how people make sense of the world; Gestalt psychology, emphasizing part-whole structures; and Phenomenology, focusing on lived experience. These influences directed research toward mapping the qualitatively different ways key educational phenomena can be experienced or understood, and arranging the variations within a part-whole structure that reflects increasing complexity of understanding [24]. While often described as a research method, phenomenography also rests on a relational epistemology that views knowledge and experience as components of the interplay between individuals and the world. In this approach, it provides a theoretical perspective for identifying the qualitatively different ways in which a phenomenon is understood within a particular group or context [24]. Phenomenography is widely used in education research exploring students’ ways of understanding a certain phenomenon, and has been used increasingly in HPE research during the last decade, hence we deem it an appropriate theoretical approach and methodology to explore how teachers understand patient involvement [24,25]. In line with the phenomenographic approach, our aim is to explore how teachers understand patient involvement in HPE, and to visualize these interrelated understandings in an outcome space. This will contribute to increased knowledge of how these understandings shape the rationales through which patient involvement is prioritized, enacted, and sustained in education.

We have chosen to use “patient” according to current practice in the field [29] and previous literature [9] acknowledging that the term comes with precautions. We use “patient” as an umbrella term and define patient as a person with a unique experience of sickness, symptoms or health care, or who is a close relative to someone who has that experience. Involvement is defined as being involved in education with the primary aim of education and not seeking care. We therefore draw a line between patient as teacher and workplace-based learning.

## Methods

The phenomenographic method is based on qualitative data, predominantly interviews. One feature of phenomenography which differs from more widely used qualitative methodologies in HPE research, is that phenomenography does not strive towards identifying common themes and categories for how a concept is experienced. Phenomenography instead aims to explore variation in ways of understanding and experiencing a concept [24,30]. Awareness of a phenomenon can be increasingly complex, the ways of understanding the phenomenon in question are therefore described in terms of expanding awareness [24,25].

In a phenomenographic study, the different ways of understanding are structured in an outcome space [24]. The differences between the categories can be explained through discernment of themes of expanding awareness within the phenomenon across the outcome space. This expansion is crucial in explaining the relationship between the categories [24]. The relationship between categories is another example of how phenomenography differs from other qualitative methods, such as reflexive thematic analysis. In phenomenography, an individual’s personal understanding is not in focus, as the methodology is used to describe the different ways in which a phenomenon can be understood by the entire dataset, in this case, the teachers [31].

### Context

The study was conducted at a medical university in Sweden with 16 HPE programs. Education takes place at two different campuses in Stockholm, several hospitals, and a plethora of public or private primary care facilities and other clinics. This study was approved by the national research ethics committee (2024-00963-01).

### Data collection

Scouting interviews were conducted with program directors and teachers from 13 of the 16 HPE programs. Teachers were defined as either faculty members or clinicians who take on a teacher role in HPE. These interviews were used to gather information to develop the interview guide and to identify eligible participants. A semi-structured interview guide was developed according to a phenomenographic approach through discussions in the research group with input from a patient representative with teaching experience. The patient representative was identified through a regional initiative from another part of Sweden focused on promoting patient involvement and lived experience in health care and education. The patient representative had no other affiliation with the university. The interview guide was pilot tested on two HPE teachers at other universities with experience of patient involvement in education. As the interviews were conducted, the guide was revisited and refined, without changing the underlying meaning of the questions.

The scouting interviews also provided information about the current state of patient involvement at the university and through these interviews, coupled with subsequent snowball sampling, twenty-five teachers with experience of patient involvement were identified and invited to participate via e-mail. Four did not respond, and one could not participate at the time of the study. Twenty teachers accepted our invitations and interviews were conducted between November 2024 and February 2025. Both written and verbal consent were acquired prior to the interview. All interviews were conducted by ES. The interviews lasted for 38–82 minutes with a mean time of 61 minutes. Thirteen interviews were conducted face-to-face on a location suggested by the interviewee and seven interviews were conducted online through Zoom. The interviews were recorded and transcribed verbatim.

### Data analysis

For the analysis of the data, we employed a 10-step guide outlined by Åkerlind and described in Table 1 [24]. As described in step 8, the process was by no means linear and included revisiting the steps repeatedly. The first six interviews were analyzed by all authors through steps 1–3, and by ES through steps 4–10, and the remaining 14 were analyzed by ES. Throughout the analytical process, iterative discussions were held between all authors about the developing results and Nvivo (version 15) was used to support the analysis.

**Table 1:** Brief description of the steps of analysis using phenomenography [24].


STEPS OF ANALYSIS	

**1**	**Familiarization** with the data. The manuscripts were read repeatedly.

**2**	**Condensing** the data by retrieving meaning-laden statements in the manuscripts. Coding of meaning-laden statement was done in Nvivo.

**3**	**Comparing** the data to identify variation and similarity. Meaning-laden statements were compared to identify variation and similarity.

**4**	**Grouping**, consists of grouping meaning-laden statements with similar meaning into categories of similar understanding of the concept. Similar meaning-laden statements were grouped together.

**5**	**Delimiting** categories from each other. Similarities within each group as well as differences between groups were clarified to distinguish categories from each other.

**6**	**Discerning** themes of expanding awareness within the different categories. Critical themes of variation describing the expanding categories were identified.

**7**	**Articulating**, includes describing the different categories and themes of expanding awareness. Categories were described by the identified themes of expanding awareness to clarify the meaning of each category and each theme.

**8**	**Checking**, emphasizes that step three to seven is an iterative process that needs to be done over and over in order to thoroughly analyze the data. Steps three to seven were done many times, iteratively.

**9**	**Labelling**, puts a short, describing label to each category and theme of expanding awareness. Each category and each theme was labelled.

**10**	**Relating**, serves to create an outcome space where the different categories of understanding the phenomenon are hierarchically structured and related to each other. Each category was related and hierarchically structured by the creation of the outcome space.


### Reflexivity

The study is part of a larger project with the aim of improving patient involvement in HPE and assisting teachers in involving patients. The research team consisted of active clinicians, teachers, and HPE researchers with experience teaching in both clinical and non-clinical settings. Several of the researchers have previous experiences with patient involvement; as students (ES, male MD, AE, female MD), as a teacher (AS, male MD), and as a researcher (TS, female HPE scholar).

As three out of the four researchers have a predominant background in the medical program and as MDs (ES, AE, AS), their experiences might have influenced the interviews and analysis of the data regarding contextual presumptions and understandings. As clinicians, educators and HPE researchers have different expertise and different perspectives on patient involvement, the authors have made varied contributions to the study development and data analysis and thus complemented each other throughout the study. This triad positioning created a solid foundation for a thorough analysis. Iterative discussions have been held throughout the study to reflect on presumptions and influence on the analysis.

## Results

The 20 respondents were teachers in eight different HPE programs (Table 2). All had experiences involving patients in their courses, inviting them to take a role either as lecturer, seminar leader, participant in a discussion group or clinical instructor. Some teachers also had experience with patient involvement in curriculum design and an informal experience of patient involvement in student assessment. Most of the participants had not introduced patient involvement in their courses but adopted it from previous teachers. All teachers were also active clinicians within their occupation.

**Table 2:** Demographic summary.


TEACHER	HPE PROGRAM	PRIMARY ROLE IN EDUCATION	NUMBER OF YEARS TEACHING IN HPE

**1**	Speech pathology program	Course director	17

**2**	Audiology program	Course director	23

**3**	Optician program	Lecturer	16

**4**	Speech therapy program	Course director	16

**5**	Medical program	Course director	12

**6**	Occupational therapy program	Clinical teacher	4

**7**	Speech therapy program	Teacher	12

**8**	Psychology program	Course director	8

**9**	Medical program	Lecturer	8

**10**	Physiotherapy program	Lecturer	6

**11**	Medical program	Course director	12

**12**	Physiotherapy program	Lecturer	15

**13**	Speech therapy program	Lecturer	5

**14**	Medical program	Clinical teacher	1

**15**	Medical program	Lecturer	>20

**16**	Psychology program	Lecturer	10

**17**	Optician program	Clinical teacher	>5

**18**	Medical program	Senior lecturer	28

**19**	Medical program	Lecturer	25

**20**	Nursing program	Clinical teacher	20


### The Outcome Space

Our data analysis constituted four hierarchical categories of understanding patient involvement. The first category is **“A way to vary education”**, mainly focusing on patient involvement as a means to make education more varied and engaging. The second category is **“A way to improve education”**, focusing on the concept that there is value added to education through patient involvement. The third category is **“A way to improve healthcare”**, seeing education as a mean of improving health care, and patient involvement as crucial to doing so. The fourth and final category of understanding patient involvement is **“A way to improve society”**, focusing on the idea that actions taken in HPE and health care affects society. These categories are differentiated from each other through a number of themes of expanding awareness, namely **the role of the patient, the role of the student, the role of the teacher, the role of education, interaction between patient and student, view of workplace learning and teachers’ perceived agency** as depicted in Table 3.

**Table 3:** The outcome space with themes of expanding awareness.


THEMES OF EXPANDING AWARENESS	CATEGORIES			

	A WAY TO VARY EDUCATION	A WAY TO IMPROVE EDUCATION	A WAY TO IMPROVE HEALTH CARE	A WAY TO IMPROVE SOCIETY

**ROLE OF THE PATIENT**	A useful means for variation of teaching methods	Has unique experiences and knowledge	Is crucial to create better healthcare	Is first and foremost a person in society

**ROLE OF THE STUDENT**	Passive	Active and complex	A future healthcare professional	Part of a larger society in which it is important to coexist

**ROLE OF THE TEACHER**	Activate the student	Improve education by sharing some teaching activities when beneficial	Create possibilities for a healthcare professional to develop	Show that society is complex and important to understand

**ROLE OF EDUCATION**	Engage students and facilitate learning through variation of teaching methods	Facilitate learning through variation, emotion and meaningfulness	Develop health care professionals and improve health care	Inspire future health care professionals to improve health care and solve problems in society

**INTERACTION BETWEEN PATIENT AND STUDENT**	One-way	Dialogue	Dialogue	Equal dialogue

**VIEW OF WORKPLACE LEARNING**	Workplace learning is better than patient involvement	Patient involvement prepares for workplace learning	Patient involvement prepares for workplace learning	Patient involvement and workplace learning are vastly different and allow for different benefits

**TEACHERS’ PERCEIVED AGENCY**	Agency to change education	Agency to change education	Agency to change the health care system	Agency to change society


### Category 1: A Way to vary Education

The first category emphasizes that much of HPE today is monotonous: the same type of lectures, followed by the same type of seminars and the same type of group exercises and workshops. Varying education is therefore important to stimulate students and keep them interested in education, implying that varied education is good education, and that patient involvement is a way to vary education that is as good as many others.

*“This thing with patient involvement, I see it as a way to run a diverse education. A course that offers different ways to learn. Actually, I don’t think I have a particular drive towards involving patients and doing that well”*

- Teacher 9

A concept that was included in this category is using the patient as a way of demonstrating a disease or symptom. Thus, creating a visual representation of, for example, a rheumatic joint or a neurological impairment. This concept emphasizes some importance of patient involvement but does not acknowledge the potential difference between patient as teacher and, for example, bedside teaching, or videos where symptoms are visually represented.

*“But I also try to include lots of pictures of patients, to show different things they can still do. But I can never explain symptom X or symptom Y as clearly as the patient can show it. Or as the patient can describe how symptom Z feels for example.”*

- Teacher 10

In general, the first category shows understanding of the patient as a valued part of the education system, and a useful means to vary education, engage students and represent symptoms and diseases. However, workplace learning can offer more clinical cases and is therefore regarded as a superior substitute to patient-as-teacher involvement. The student is portrayed as passive, and in need of stimulation and variation in teaching methods to achieve learning, while the teacher’s role is to create a stimulating environment with agency to vary education through patient involvement, but not to create larger scale improvements or change.

### Category 2: A Way to improve Education

While including all aspects from Category 1, the second category expands the understanding of the role patients may take in education, adding the patient’s history to the representation of symptoms and disease, thus including the patient’s own perspective. In this category, much of the focus is on the emotional aspect of encountering a patient, listening to their history and thus, enhancing knowledge and memory creation from emotional stimulation.

*“If you look at Aristoteles ethos, logos and pathos, we mainly touch upon pathos. And from my own experience in education, when you create an emotion in someone, the possibility to learn is much greater. And if you look back at your career, at what you have done, you often remember the situations that were a bit more emotional.”*

- Teacher 18

Furthermore, teachers in the second category perceived patient involvement to be a good complement to workplace learning in general, and especially as preparation for clinical placements. Interviewees described that patient involvement allowed more time and focus on understanding the patient, as well as an opportunity for dialogue, which was seen as beneficial for the more stressful subsequent clinical placements.

*“…it is more like this, ‘okay, this is how a patient can be like’. When you just read about it, it can sound a bit scary and difficult… Then when you’re on your clinical placements or at your workplace in the future, it’s not as scary.”*

- Teacher 13

Another aspect added in this category was the aspect of dialogue with the patient, implying that there is additional value created in meeting and talking with the patient. Partly because it shows that all patients and all meetings are unique, but also because dialogue creates understanding and allows a larger part of the patient’s perspective to be shown.

*“It [a video clip] isn’t as alive, I think. It gets a lot more fragmented if you find it on YouTube or something like that. And first and foremost, there’s no possibility for dialogue.”*

- Teacher 11

Regarding how the patient is described, there is expansion from the first category, as the patient’s experiences are considered increasingly valuable here to create emotion and meaning to education. They are also valued as teachers, although not equally valued as academic teachers. The student is seen as complex, interested, and can interact with the patient while the teacher shares the role as teacher with the patient in a controlled manner where patient involvement can be implemented to add meaning, emotion, or to connect theory with practice. Thus, improving quality in education, indicating a perceived agency for the teacher to not only vary, or create change in the classroom, but create change to education in general.

### Category 3: A Way to improve Healthcare

Expanding on Category 2, Category 3 is characterized by positioning education in relation to the health care system and emphasizing the link in between, meaning that the role of education is to train future health care professionals. As in Category 2, the focus is on the patient’s perspective and history, which in Category 3 is also placed in the context of health care, and person-centeredness in health care is suggested as a goal of patient involvement in HPE.

*“I think [it is important] to always have that in the back of your head, that there is so much more you need to know than the obvious that comes with the referral. You have to understand all of it. Because I believe that will help you understand why patient A reacts to a treatment in one way, and patient B in another way.”*

- Teacher 8

As in Category 2, the emotional part of patient involvement in education is mentioned in Category 3, but an additional emotion is that of inspiration. Inspiration can work in different ways, either through inspiring the student to work in health care or inspiring a student to choose a specific career path or by showing the positive aspect of the patient’s experience. This positivity can be either through displaying how well health care can work, or by demonstrating that the patient, despite suffering from a disease, can live a fulfilling and meaningful life.

*“These patients are severely affected. ‘Will I be able to deal with this as a doctor?’ I want something where you start a treatment and the patient feels well. But these patients, almost everyone is severely affected by their disease. And then you hear one say: ‘I still work full time, and next month, I’m getting married.’ Or the opposite, divorces and so on. I mean, life goes on, even if you have a disease.”*

- Teacher 19

The notion that the patient’s life outside of the health care system also is of importance in the provision of good care is an expanded awareness of the role of the patient in this category. The patient’s perspective is seen as crucial to understanding this concept, and therefore irreplaceable. In this category, there is an understanding that the students are future health care professionals and that there is a connection between understanding the patient as a person and working with qualitative person-centered care. There is also a description of problems within health care that patient involvement can solve, mainly that the lack of person-centeredness needs more, and earlier interventions, but also that understanding certain patient groups is crucial to solve the problem of unequal care towards marginalized groups.

*“Yes, these are very serious problems. And that is what we’re talking about, stigma. Many patient groups suffer from this on a common basis. I can imagine it happens to people from different cultures as well. That you have presumptions about the fact that they seek care on some occasions, I mean, there’s stigma towards… some patients, I believe, very often receive bad treatment.”*

- Teacher 11

Teacher 11 perceived patient involvement as a way to reduce the stigma and improve health care for marginalized groups. This quote symbolizes the perceived agency that patient involvement can improve health care for future patients. Therefore, Category 3 includes the idea that teachers perceive themselves as being able to affect the health care system with their teaching. Furthermore, the role of education is emphasized as crucial to improving health care, while the role of the student in this category is positioned in relation to their future roles as health care professionals.

### Category 4: A Way to improve Society

Expanding further on the understanding of patient involvement, the health care system and the context in which teachers, patients and students operate, Category 4 includes an understanding that both education and healthcare affect, and are affected by, the society in which they exist. The importance of education is therefore not limited to health care, but perceived as a means for improving society in general. This way of understanding patient involvement in education also emphasizes perspectives of patients and students from outside the healthcare system. Therefore, as Teacher 8 suggests, it is important to be genuinely interested and curious about the person in front of you, not only as a health care professional, but as a person with whom to engage in equal dialogue.

*“An interest in people actually. It might sound weird but I think that instead of only having course literature and try to understand why it’s important to do in a certain way, my hope is that it’s more like this: ‘This is super interesting. It’s really interesting to talk to a person who has had X. It’s not scary. They’re not dangerous. They’re no different from you and me.”*

- Teacher 8

This quote points to an expansion in awareness within Category 4, that patient involvement may raise questions about equality and normality within society. Thus, involvement can illustrate that a patient might be treated differently, not only within healthcare, but in society in general, because their disease or symptom is not considered normal.

*“It can be interesting to talk about things like normality […] That it’s interesting to discuss what is normal and what is a deviation. What do we classify as normal and where do we draw the line?”*

- Teacher 4

In Category 4, there is a perception that involvement in education empowers the patient, who in this category not only affects education and healthcare by engaging in education but also affects society in a wider sense. The patient in this category is no longer “used” in education, the teachers perceive that the patient meets the teacher halfway and shares responsibility in education. Consequently, teachers perceive that patient involvement is a possibility for the patient to engage in education, healthcare, and society, and to improve it.

*“But they also feel that what they do is important, an important thing, to spread awareness of, to make it generally better for people with symptom X, that’s a bonus.”*

- Teacher 7

This category suggests that patient involvement in education may not only solve problems in healthcare, but in society in general, expanding on the problem formulating part of Category 4 to also include problems in society. This inclusion implies that teachers perceive patient involvement as being a means for patient empowerment and emancipation, through giving the patients power to improve their own status in society. Category 4 also acknowledges that the patient’s role in this context may differ from that in workplace learning. Both types of involvement are, however, important to education. The following quote represents one of the ways in which patient involvement can differ from workplace learning; that it allows for the student to let go of prestige and focus less on being a good student and more on being a person interested in another person.

*”There is no prestige in this… More than asking and being active in the conversation. But you’re not supposed to solve any problems. Because as a student you’ve got the idea that you’re supposed to display your knowledge and show your skills, that’s why they’re here and that’s all good, but in this case it’s more about being a person.”*

- Teacher 20

Furthermore, the teachers’ perceived agency in this category includes not only the capacity to change education, or health care, but society in general. In this way, it indicates an understanding that involving patients in HPE touches upon questions that extend beyond both education and health care.

## Discussion

This study set out to explore how teachers understand patient involvement in HPE. Our findings show that these understandings vary in both perspective and scope, reflecting different perceptions about the purpose and influence of patient involvement. The understandings range from viewing patient involvement as a way to enrich teaching to seeing it as a means to improve health care and even contribute to societal change. This progression forms the central finding of our study, highlighting how teachers’ understandings expand in breadth and ambition.

In the less complex categories, patient involvement is understood as something that can enrich teaching within the classroom, and teachers describe their influence as limited to shaping educational activities. As understandings expand, so does the teachers’ sense of what they are able to affect: first the quality of education, then aspects of health care, and finally broader social conditions. This suggests that teachers perceive patient involvement as a mean for change, a view that aligns closely with the concept of agency—often defined as “the capacity to produce an effect” and previously identified as central in HPE [26,27].

However, patient involvement is rarely an activity that teachers accomplish alone. It depends on relationships between teachers, students, patients, and the wider educational environment. This also means that agency in patient involvement should not be understood solely as an individual capacity; it is affected by what others in the system are able and willing to do. The concept of relational agency captures this dynamic: a capacity to align one’s actions and perceptions with those of others in order to respond to shared problems of practice [27,28]. In the context of patient involvement, relational agency becomes especially important, as meaningful inclusion requires coordinated action between teachers and patients, and often relies on the patient’s own motivations, experiences, and sense of influence.

Previous research has shown that patient involvement can function as a form of emancipation for patients, providing opportunities to influence how future professionals understand illness, care, and the patient role [23]. In our study, this emancipatory potential appears most clearly in the more complex categories, where teachers perceive patient involvement not only as a pedagogical resource but as a way for patients to contribute to health care and society. In contrast, the less complex categories reflect a narrower view, where patient involvement is valued for providing variation in teaching methods, but less connected to patient influence. This finding suggests that more complex understandings may better align with patients’ own aspirations for involvement, creating conditions in which agency is shared and negotiated rather than held primarily by teachers.

**Figure 1:**
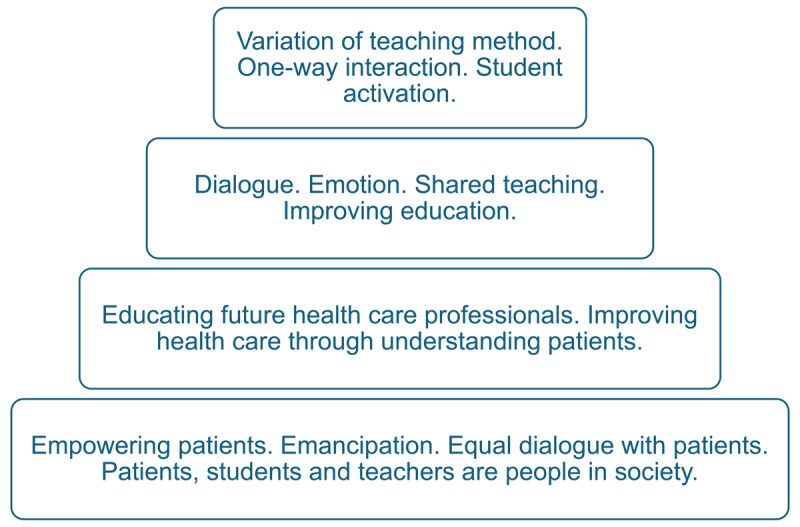
The layered cake metaphor visualizing that a more complex understanding is crucial as a sustainable foundation for patient involvement in HPE.

To illustrate how the different understandings identified in our findings relate to one another, we propose a layered cake metaphor (Figure 1). The most complex category forms the foundational layer, providing stability and direction for more sustainable forms of patient involvement. The least complex category can be seen as the icing—adding value and variation, but unlikely to support deeper or systemic change on its own. All categories contribute beneficial elements, but when striving for change, shared power, or emancipation, we believe that more complex understandings form the necessary base.

Teachers’ understandings of patient involvement are affected by the organizational and structural conditions in which they work. Several teachers described how patient involvement is often de-prioritized when time or financial resources are reduced. Such cutbacks limit not only the practical opportunities to involve patients but also teachers’ sense of agency, as their capacity to influence teaching becomes more constrained. Beyond economic and temporal factors, social structures, such as habitual teaching practices, expectations about what constitutes appropriate teaching and learning practices, and implicit assumptions about patients’ roles, also appear to shape how teachers perceive patient involvement. This finding highlights that teachers’ understandings do not develop in isolation but emerge in dialogue with the structural affordances and constraints of their educational environment.

Taken together, our findings suggest that supporting teachers in developing more complex understandings of patient involvement may be crucial for promoting sustainable and meaningful engagement of patients in education. When patient involvement is seen primarily as a way to add variation to teaching, it may be vulnerable to organizational pressures and less likely to foster shared agency between teachers and patients. In contrast, when patient involvement is understood as contributing to improved education, health care, and society, teachers may be better equipped to articulate its value, negotiate for its inclusion, and collaborate with patients in ways that align with their goals and experiences. Educational developers, programme leaders, and institutions may therefore need to create structures that support such developments, for example by providing opportunities for reflection, collaboration with patients, and institutional recognition of the value of patient involvement.

### Methodological considerations

This study used a phenomenographic approach to explore how teachers understand patient involvement in HPE. Phenomenography was well suited to this aim, as it focuses on variation in experience and the structural relationships between different ways of understanding.

Although health professional, educator and HPE scholar perspectives were represented in the research group, it consisted predominantly of MDs and a more diverse research group might have been better suited for analysis of the wide spectrum of HPE programmes. Whilst patient perspective informed the study design, we acknowledge we could have included the patient perspective to a greater extent in the study design.

Teachers from eight HPE programmes participated as respondents, providing a broad set of experiences and strengthening the transferability of the findings. All teachers in all the programmes that had experience of patient involvement were invited to participate, resulting in 20 respondents, which is consistent with phenomenographic research and adequate given the study’s information power, supported by the specificity of the aim and the richness of the interviews [32]. We acknowledge the tension between researching patient involvement without including patients as respondents. However, teachers are key stakeholders in implementing patient involvement, and understanding their perspectives is a necessary step.

### Conclusions and future Implications

Our study demonstrates considerable variation in how teachers understand patient involvement in HPE, ranging from viewing patients as a means to vary teaching to seeing them as contributors to improvements in education, health care, and society. Recognizing and addressing this variation is essential for strengthening patient involvement in HPE, as teachers’ perceptions influence the opportunities they create for patients and the value they attribute to their contributions.

The findings suggest that more complex understandings of patient involvement may support forms of engagement that are pedagogically effective, sustainable, and better aligned with patients’ perspectives. Teachers who see patient involvement as part of a broader educational, clinical, and societal endeavor may be better positioned to negotiate for its inclusion and defend it under organizational pressures. This further underscores the importance of institutional structures and faculty development that create opportunities for reflection and for meaningful collaboration between teachers and patients.

Future research should examine how different stakeholders’ understandings of patient involvement interact in practice, particularly given the relational nature of agency. Understanding how teachers’, patients’, and students’ perspectives align, or fail to align, may shed further light on the conditions required for meaningful and sustainable patient involvement.
